# SARS-CoV-2 infection impairs NK cell functions *via* activation of the LLT1-CD161 axis

**DOI:** 10.3389/fimmu.2023.1123155

**Published:** 2023-05-23

**Authors:** Marzena Lenart, Magdalena Górecka, Michal Bochenek, Emilia Barreto-Duran, Artur Szczepański, Adrianna Gałuszka-Bulaga, Natalia Mazur-Panasiuk, Kazimierz Węglarczyk, Andżelika Siwiec-Koźlik, Mariusz Korkosz, Paweł P. Łabaj, Monika Baj-Krzyworzeka, Maciej Siedlar, Krzysztof Pyrc

**Affiliations:** ^1^ Virogenetics Laboratory of Virology, Malopolska Centre of Biotechnology, Jagiellonian University, Krakow, Poland; ^2^ Flow Cytometry Core Facility, Malopolska Centre of Biotechnology, Jagiellonian University, Krakow, Poland; ^3^ Department of Clinical Immunology, Institute of Pediatrics, Jagiellonian University Medical College, Krakow, Poland; ^4^ Department of Rheumatology and Immunology, Jagiellonian University Medical College, Krakow, Poland; ^5^ 2nd Department of Internal Medicine, University Hospital, Krakow, Poland; ^6^ Bioinformatics Research Group, Malopolska Centre of Biotechnology, Jagiellonian University, Krakow, Poland

**Keywords:** NK cells, SARS-CoV-2, CD161-LLT1 axis, NK cell impairment, antiviral response

## Abstract

**Introduction:**

Natural killer (NK) cells plays a pivotal role in the control of viral infections, and their function depend on the balance between their activating and inhibitory receptors. The immune dysregulation observed in COVID-19 patients was previously associated with downregulation of NK cell numbers and function, yet the mechanism of inhibition of NK cell functions and the interplay between infected cells and NK cells remain largely unknown.

**Methods:**

In this study we show that SARS-CoV-2 infection of airway epithelial cells can directly influence NK cell phenotype and functions in the infection microenvironment. NK cells were co-cultured with SARS-CoV-2 infected epithelial cells, in a direct contact with A549^ACE2/TMPRSS2^ cell line or in a microenvironment of the infection in a 3D ex vivo human airway epithelium (HAE) model and NK cell surface expression of a set of most important receptors (CD16, NKG2D, NKp46, DNAM-1, NKG2C, CD161, NKG2A, TIM-3, TIGIT, and PD-1) was analyzed.

**Results:**

We observed a selective, in both utilized experimental models, significant downregulation the proportion of CD161 (NKR-P1A or KLRB1) expressing NK cells, and its expression level, which was followed by a significant impairment of NK cells cytotoxicity level against K562 cells. What is more, we confirmed that SARS-CoV-2 infection upregulates the expression of the ligand for CD161 receptor, lectin-like transcript 1 (LLT1, CLEC2D or OCIL), on infected epithelial cells. LLT1 protein can be also detected not only in supernatants of SARS-CoV-2 infected A549^ACE2/TMPRSS2^ cells and HAE basolateral medium, but also in serum of COVID-19 patients. Finally, we proved that soluble LLT1 protein treatment of NK cells significantly reduces *i)* the proportion of CD161+ NK cells, *ii)* the ability of NK cells to control SARS-CoV-2 infection in A549^ACE2/TMPRSS2^ cells and *iii)* the production of granzyme B by NK cells and their cytotoxicity capacity, yet not degranulation level.

**Conclusion:**

We propose a novel mechanism of SARS-CoV-2 inhibition of NK cell functions via activation of the LLT1-CD161 axis.

## Introduction

Coronavirus disease 2019 (COVID-19), caused by severe acute respiratory syndrome coronavirus 2 (SARS-CoV-2), was diagnosed in over half a billion people and confirmed as the cause of death of over 6.3 million globally (1). These numbers however, are estimated to be heavily understated, and the approximated number of deaths caused by COVID-19 may be roughly four times higher (2). The clinical outcome of the disease ranges from mild respiratory tract infection to severe life-threatening illnesses manifesting in the form of acute respiratory distress syndrome (ARDS) and cytokine storm syndrome (CSS) (3). While COVID-19 is primarily a respiratory system disease, SARS-CoV-2 virus possess the ability to spread systematically and infect several tissues in the human body (4).

Viral infections are largely controlled by natural killer (NK) cells as part of the innate immune response. NK cells are a large, granular lymphocytes, and their content in the peripheral blood falls between 5 and 20% (5). NK cells functions are regulated by the balance between their germ-line encoded surface receptors, which can be categorized as either activating (CD16, NKG2D, NKp30, NKp44, NKp46, DNAM-1 NKG2C, killer cell immunoglobulin-like receptors [KIRs]) or inhibitory (particular KIRs, NKG2A, TIGIT, Tim-3, or PD-1) (6–10). NK cell-mediated killing of infected or neoplastic cells occurs *via* direct lysis of target cells by perforin (Prf) and granzymes secreted from NK cell lysosomes through activation of Fas- and tumor necrosis factor (TNF)-related apoptosis-inducing ligand (TRAIL)-associated apoptosis (11). NK cells can also secrete a number of cytokines and chemokines, such as interferon γ (IFN-γ), TNF-α, granulocyte-macrophage colony-stimulating factor (GM-CSF), interleukin 5 (IL-5), IL-13, macrophage inflammatory protein (MIP-1) and RANTES (12).

The role of NK cells during COVID-19 is still extensively studied, but most reports focus solely on clinical data. The immune dysregulation observed in COVID-19 patients is associated with downregulation of NK cell numbers and function. Significantly impaired NK-cell counts and cytolytic activity were observed in severe COVID-19 patients when compared with healthy controls (13); patients with normal NK cell counts were reported to exhibit a faster decline in viral load than patients with low NK numbers (14). High IL-6 plasma levels, characteristic of severe-to-critical COVID-19 patients, were associated with the suppression of IFN-γ production by NK cells and inhibition of NK cell activity (14). Increasing evidence suggests that alterations of NK cell receptors, both activating and inhibitory, might contribute to the dysfunctional status of COVID-19-associated NK cells. Previous reports detected higher levels of NKG2A, PD-1, and CD39 in COVID-19-associated NK cells from the peripheral blood and bronchoalveolar lavage fluid (15). NK cells contact with SARS-CoV-2 Spike protein subunit S1 artificially expressed on lung epithelial cells resulted in NK cell exhaustion *via* HLA-E/NKG2A interaction (16), whereas simultaneous blockade of all three natural cytotoxicity receptors (NKp30, NKp44, and NKp46) or 2B4, NKG2D and DNAM-1 led to a significant increase in virus replication, suggesting that NK-cell-mediated control of SARS-CoV-2 replication requires redundant recognition by NK cell receptors (17). However, mechanisms of NK cell function inhibition and the interplay between infected cells and NK cells remain to be fully understood.

CD161 (NKR-P1A or KLRB1) is a C-type, lectin-like glycoprotein expressed on human NK cells and T cells (18), which binds to lectin-like transcript 1 (LLT1, CLEC2D or OCIL), its only known ligand broadly expressed on cells of the hematopoietic lineage (19). Contrasting with CD161 on T cells, which has been shown to enhance cytokine production following TCR triggering, CD161 on NK cells appears to act as an inhibitory receptor (10, 20), yet little is known about its role in NK cell antiviral response. CD161 marks NK cells that have retained the ability to respond to innate cytokines during their differentiation, and is lost upon cytomegalovirus-induced maturation in both healthy and HIV-infected patients (21). On the other hand, transfection of LLT1 into NK cell targets protects them from human NK cell cytotoxicity in a CD161-dependent manner (22).

This study shows that SARS-CoV-2 infection of epithelial cells can directly influence NK cell functions in the infection microenvironment. During infection, an extensive amount of the LLT1 protein is released from the infected tissues, which in turn binds to its receptor, CD161, expressed on the surface of NK cells. Due to the ligand-receptor interactions, the observed CD161 surface expression is downregulated, while NK cell functions and virus clearance are impaired. Consequently, we propose a new mechanism of NK cell function modulation during the SARS-CoV-2 infection *via* activation of the LLT1-CD161 axis.

## Materials and methods

### Cell lines and culture

Vero (*Cercopithecus aethiops*; kidney epithelial; ATCC CCL-81), and A549 (ATCC: CCL-185) expressing ACE2 and TMPRSS2 (A549^ACE2/TMPRSS2^, generated *in-house* (4)) were maintained in Dulbecco’s minimal essential medium (DMEM; Gibco, Thermo Fisher Scientific, Waltham, MA, USA) supplemented with 5% v/v heat-inactivated fetal bovine serum (FBS; Gibco, Thermo Fisher Scientific) and penicillin-streptomycin solution (100 U/ml and 100 µg/ml, respectively) (PAN Biotech GmbH, Bayern, Germany). Medium for A549^ACE2/TMPRSS2^ cells was additionally supplemented with puromycin (0.5 μg/ml, EPRO, Poland) and blasticidin S (10 µg/ml; Sigma-Aldrich, Merck, Warsaw, Poland). K562 cells (human erythroleukemic cell line; ATCC CCL-243) were cultured in RPMI 1640 medium (Gibco), supplemented with 5% v/v heat-inactivated FBS, penicillin-streptomycin solution (100 U/ml and 100 µg/ml, respectively) and 1x Glutamax (Gibco).

Primary human bronchial epithelial cells were purchased (Epithelix Sarl, Geneva, Switzerland) and expanded in the bronchial epithelial growth medium (BEGM) in-house. When confluent, cells were detached using trypsin and seeded onto permeable Thincert™ culture inserts (Greiner Bio-One, Kremsmünster, Austria). Cells were cultured submerged in a BEGM medium on the apical and basolateral side until confluent; then the apical medium was discarded, while the basolateral medium was changed to an air-liquid interface (ALI) medium. Cells were cultured for 4 weeks to form fully-differentiated, polarized cultures that manifested *in vivo* pseudostratified mucociliary epithelium phenotype (human airway epithelium cultures, HAE). Cells were maintained at 37°C under 5% CO_2_.

### Virus strain

The following virus strain was used: SARS-CoV-2 PL_P18 [GISAID Clade G, Pangolin lineage B.1] (accession numbers for the GISAID database: EPI_ISL_451979). SARS-CoV-2 stocks were generated by infecting monolayers of Vero cells. The cells were maintained at 37°C under 5% CO_2_ and the virus-containing liquid was collected on day 3 post-infection (p.i.), aliquoted, and stored at −80°C. Control samples from mock-infected cells were prepared correspondingly. Virus yields were assessed by titration on fully confluent cells in 96-well plates, according to the method of Reed and Muench (23). Plates were incubated at 37°C, and the cytopathic effect (CPE) was scored by observation under an inverted microscope.

### COVID-19 patients

For this study, we recruited patients with SARS-CoV-2 pneumonia (n=11) hospitalized at the University Hospital, Cracow, between September 5^th^, 2020, and February 2^nd,^ 2022. Diagnosis of SARS-CoV-2 was based on the nasopharyngeal swab results demonstrating positivity by reverse transcriptase-polymerase chain reaction (RT-PCR). Enrollment criteria included: age >18 years, hospital admission, positive SARS-CoV-2 RT-qPCR result, respiratory symptoms (cough, shortness of breath), fever, imaging showing ground glass opacities in the lungs by chest roentgenogram or CT, and informed consent. Treatment for COVID-19 was permitted, according to local clinical practice. Patients were studied during the first two weeks after hospital admission. Mean age (± SD) of patients was 61 ± 11, the group consisted of six men and 5 women. Age-matched healthy subjects (n=10) were considered a reference for the investigational analyses. Mean age (± SD) of control group was 52 ± 9, the group consisted of two men and eight women.

This study was approved by the Local Bioethics Committee (78/KBL/OIL/2020) and complied with the Declaration of Helsinki and good clinical practice guidelines. All participants provided signed informed consent.

### NK cell isolation

Anticoagulated citrate dextrose-A-treated blood from healthy donors was purchased from the Regional Centre of Blood Donation and Blood Therapy in Krakow, Poland. Peripheral blood mononuclear cells (PBMC) were isolated by the standard density gradient centrifugation using Pancoll human (Panbiotech, Aidenbach, Germany). Lymphocytes were then separated from PBMC with the AVANTI J-26S XP elutriation system, equipped with the Sanderson separation chamber (Beckman Coulter, Brea, CA, USA), as described previously (24). NK cells were isolated from leukocytes using MACS technology and a Dynabeads™ Untouched™ Human NK Cells Kit (Invitrogen, Thermo Fisher Scientific, Waltham, MA, USA), in which NK cells were isolated *via* negative selection. NK cells were then cultured in LGM-3 medium (Lonza, Basel, Switzerland) supplemented with 5% v/v heat-inactivated human AB serum (Sigma, St. Louis, MO, USA), 500 U/ml IL-2 (STEMCELL Technologies), 140 U/ml IL-15 (R&D Systems, Minneapolis, MN, USA), and penicillin-streptomycin solution (100 U/ml and 100 µg/ml, respectively) (PAN Biotech GmbH, Bayern, Germany). Each experiment was performed using NK cells isolated from a different blood donor.

### Viral infection

Cells were infected for 2h at 37°C with SARS-CoV-2 with a TCID_50_ of 800/ml for Vero cells, TCID50 of 1600/ml for A549^ACE2/TMPRSS2^ cells or mock-treated with the medium collected from uninfected cellsAfter 2h infection, cultures were washed thrice with phosphate-buffered saline (PBS, Gibco) and cultured in DMEM supplemented with 5% v/v heat-inactivated fetal bovine serum (FBS).

HAE cultures were infected apically for 2h at 37°C with SARS-CoV-2 at 5000 TCID_50_/ml, or mock-treated. After infection, cultures were washed thrice with PBS, and the third PBS wash was collected as a first (2 h p.i.) sample. Cultures were left at an ALI for the remaining time of the experiment. Later, samples were collected every 48 or 72h until 120h p.i. For sample collection, PBS (100 µl) was added to the apical side of the inserts and incubated for 15 minutes at 37°C; then, it was collected for RT-qPCR analysis.

### NK cell co-cultures with epithelial cells

NK cells were added to monolayers of Vero or A549^ACE2/TMPRSS2^ cells, 24 or 48 h p.i. Co-cultures were maintained in LGM-3 medium (Lonza) supplemented with 5% v/v heat-inactivated human AB serum (Sigma), 500 U/ml IL-2 (STEMCELL Technologies), 140 U/ml IL-15 (R&D Systems), and penicillin-streptomycin solution (100 U/ml and 100 µg/ml, respectively) (PAN Biotech GmbH) for 24h. In the case of HAE cultures, NK cells in LGM-3 medium (Lonza) supplemented with 5% v/v heat-inactivated human AB serum (Sigma), 500 U/ml IL-2 (STEMCELL Technologies), 140 U/ml IL-15 (R&D Systems), and penicillin-streptomycin solution (100 U/ml and 100 µg/ml, respectively) (PAN Biotech GmbH) were added to the basolateral side of HAE and maintained for 72 h.

### Isolation of nucleic acids and RT-qPCR

To monitor the infection in Vero and A549 ^ACE2/TMPRSS2^ cell lines, the viral RNA was isolated from infected cell cultures. The supernatants and cell lysates were collected and processed using a Viral DNA/RNA Kit (A&A Biotechnology, Gdansk Poland), according to the manufacturer’s instructions. The viral RNA was quantified using GoTaq^®^ Probe 1-Step RT-qPCR System (Promega, Madison, WI, USA), specific SARS-CoV-2 N gene probe (200 nM, 5’-ACT TCC TCA AGG AAC AAC ATT GCC A-3’ (FAM/BHQ1)), and primers (Forward: 600 nM, 5’-CAC ATT GGC ACC CGC AAT C-3’, Reverse: 800 nM, 5’-GAG GAA CGA GAA GAG GCT TG-3’). In order to assess the copy number for the N gene, standards were prepared and serially diluted. The heating scheme was: 15 min at 45°C and 2 min at 95°C, followed by 40 cycles of 15s at 95°C and 1 min at 58°C or 60°C. RT-qPCR reactions were performed in CFX96 Touch Real-Time PCR Detection System (Biorad, Hercules, CA, USA).

### Flow cytometry

The following mouse monoclonal antibodies (mAbs) against human molecules were used: anti-CD16-FITC (clone CB16, Invitrogen), anti-CD16-PE-Cy7 (clone B73.1, BD Biosciences, Franklin Lakes, NJ, USA), anti-CD56-PE-Cy5 (clone CMSSB, Invitrogen), anti-CD56-PE-Cy7 (clone NCAM16.2, BD Biosciences), anti-CD3-PerCP (clone SK7, BD Biosciences), anti-CD14-APC (clone MϕP9, BD Biosciences), anti-NKG2D-PE (clone 1D11, Invitrogen), anti-NKp46-PE-Cy7 (clone 9E2, Invitrogen), anti-DNAM-1-APC (clone 11A8.7.4, Invitrogen), anti-NKG2A-PE (clone REA110, Miltenyi Biotech, Bergisch Gladbach, Germany), anti-TIGIT-PE-Cy7 (clone MBSA43, Invitrogen), anti-TIM-3-APC (clone F38-2E2, BioLegend, San Diego, CA, USA), anti-PD-1-PE-Cy7 (clone EH12.1, BD Biosciences), anti-NKG2C-APC (clone 134591, R&D Systems), anti-CD161-APC (clone HP-3G10, BioLegend), anti-CD161-Alexa Fluor 700 (clone HP-3G10, BioLegend), anti-Granzyme B-PE (clone GB11, Invitrogen), perforin-PE (clone δG9, BD Biosciences), anti-TNF-APC (clone Mab11, Invitrogen) and anti-IFN-γ-PE-Cy7 (clone 4S.B3, Invitrogen).

The surface molecules were labeled by incubating NK cell samples with mAbs for 20 min at 4°C. Next, samples were washed twice with PBS, fixed with 3.7% paraformaldehyde (PFA, Sigma-Aldrich) solution in PBS, washed and resuspended in PBS, and analyzed on a Navios flow cytometer (Beckman Coulter).

Intracellular staining of granzyme B, perforin, TNF, and IFN-γ was performed 18 h post-stimulation of NK cells with a 1× cocktail of phorbol 12-myristate 13-acetate (PMA) and ionomycin (Cell Stimulation Cocktail, eBioscience, Thermo Fisher Scientific, San Diego, CA, USA) in the presence of 1× cocktail of Brefeldin A and Monensin (Protein Transport Inhibitor Cocktail, eBioscience). First, the cells were fixed with 3.7% PFA in PBS and permeabilized with 0.1% Tween-20 in PBS for 20 min at 4°C. Then, the cells were washed twice with PBS and incubated with mAbs for intracellular staining for 30 min at 4°C. The cells were washed twice, resuspended in PBS, and analyzed on a Navios flow cytometer (Beckman Coulter).

SARS-CoV-2 N protein intracellular expression cells were detached from a culture surface using 0.05% Trypsin/EDTA solution (Gibco). Cells were washed with DMEM supplemented with 5% v/v heat-inactivated FBS and then with PBS, after which 3.7% PFA solution in PBS was added for 30 min. Next, cells were washed with PBS, permeabilized with 0.5% Tween-20 in 1×PBS for 15 min at 4°C, washed again in PBS, and incubated with primary Ab, rabbit anti-SARS CoV-2 Nucleocapsid Ab (Invitrogen; 1:200 in PBS, 1h, room temperature (RT)) followed by the goat anti-rabbit Alexa Fluor 488 secondary antibody (Invitrogen; 1:200 in PBS, 30min, RT). Finally, cells were washed, resuspended in PBS, and analyzed on a Navios flow cytometer.

For all flow cytometry analyses, appropriate isotype-matched controls were included, or Fluorescence Minus One (FMO) control was used. For the detection of infected cells, negative control consisted of a mock-infected cell sample. Data analysis was performed using Kaluza Analysis software (Beckman Coulter). The expression of analyzed molecules was presented as a percentage of positively stained cells and the mean fluorescent intensity (MFI).

### NK cell sorting

Lymphocytes were stained for surface molecules with the following mAbs: anti-CD3-FITC, anti-CD16-PE-Cy7, anti-CD56-PE-Cy7, and anti-CD161-APC, as described above, and then sorted using CytoFLEX SRT Benchtop Cell Sorter (Beckman Coulter). Sorted cells were collected into FBS-coated polypropylene tubes (BD Biosciences). The purity of sorted cell populations exceeded 95%.

### NK cell cytotoxicity assay

K562 cells were labeled with 5-(and 6)-Carboxyfluorescein diacetate succinimidyl ester (CFSE; CFSE Cell Division Tracker Kit, BioLegend) according to the manufacturer’s protocol. NK cells were then added to CFSE-labelled K562 cells in a ratio of 5:1 and incubated for 4 h at 37°C under 5% CO_2_. After this time, cells were stained with fixable LIVE/DEAD dye (LIVE/DEAD™ Fixable Violet Dead Cell Stain Kit, for 405 nm excitation, Invitrogen) or 7-Amino-Actinomycin D (7-AAD; Beckman Coulter), according to the manufacturer’s protocol and then fixed with 3.7% PFA in PBS and washed in 1×PBS, or non-fixed when 7-AAD was used, and analyzed on Navios flow cytometer.

### NK cell degranulation assay

NK cells were added to K562 cells in a ratio of 5:1, along with anti-CD107a-APC mAB (clone H4A3, BioLegend) or IgG1-APC (BioLegend) and incubated for 1 h at 37°C under 5% CO_2_. Then, PMA and ionomycin (Cell Stimulation Cocktail, eBioscience, Thermo Fisher Scientific) was added along wth 1× cocktail of Brefeldin A and Monensin (Protein Transport Inhibitor Cocktail, eBioscience), and the cells were incubated 4 h at 37°C under 5% CO_2_ and analyzed on flow cytometer.

### Immunostaining for confocal microscopy

A549^ACE2/TMPRSS2^ cells cultured on glass slides and HAE cultured on the inserts with membranes were fixed with 3.7% PFA for 1h and washed with 1×PBS. Next, samples were permeabilized with 0.5% Triton X-100 in PBS for 20 min (HAE) or 0.5% Tween-20 in PBS for 15 min (A549 cells) at RT and then washed with PBS. A549^ACE2/TMPRSS2^ cells and HAE were blocked with 5% and 10% bovine serum albumin (Sigma Aldrich) in PBS, respectively (37°C, 1h). After this time, the blocking mixture was discarded, and the cells were washed thrice with PBS. Primary antibodies (**Table 1**) in 1% (A549) or 5% (HAE membranes) BSA in PBS were added and incubated with the samples for 2h at RT. Samples were washed thrice with 0.5% Tween 20 in PBS. Secondary antibodies (**Table 1**) in PBS were added and incubated for 2h at RT. After this, samples were washed thrice with 0.5% Tween 20 in PBS. The samples were incubated with 4’,6-diamidyno-2-fenyloindol (DAPI, 0.1µg/ml; Sigma Aldrich) for 20 min at RT. Samples were washed with PBS, and Alexa Fluor™ 647 Phalloidin (Invitrogen) was added at 1:400 dilution in PBS, and samples were incubated for 1 hour at RT in the dark. The slides and membranes were washed with PBS. In the case of the insert membranes, the membranes were detached from the insert with a scalpel. Both preparations were mounted to the glass slides in ProLong™ Diamond Antifade Mountant (Invitrogen) and analyzed on Carl Zeiss, ZEN 2012 SP1, LSM 710 confocal microscope (Carl Zeiss Microscopy GmbH, Jena, Germany).

**Table 1:** List of Abs used for immunostainings for confocal microscopy.

Antibody	Host species	Dilution and final concentration	Company
Anti-SARS CoV-2 Nucleocapsid	Rabbit	1:200 (5 µg/ml)	Invitrogen
OCIL/CLEC2d Antibody (4C7)	Mouse	1:200 (2.5 µg/ml)	Novus Biologicals, Bio-Techne, Centennial, CO, USA
Anti-rabbit Alexa Fluor 546Secondary antibody	Goat	1:400 (5 µg/ml)	Invitrogen
Anti-mouse Alexa Fluor 488Secondary antibody	Donkey	1:400 (5 µg/ml)	Invitrogen
Rabbit IgG isotype control	Rabbit	1:2000 (2.5 µg/ml)	GeneTex, Irvine, CA, USA
Mouse IgG isotype control	Mouse	1:2000 (2.5 µg/ml)	GeneTex, Irvine, CA, USA

### LLT1 ELISA assay

LLT1 concentration in cell culture supernatants was analyzed using Human CLEC2D ELISA Kit (Fine Test, Wuhan, China), according to manufacturer’s protocol. The analysis was performed in A549^ACE2/TMPRSS2^ cell supernatants and HAE basolateral medium, collected straight before the addition of NK cells, that is 72h p.i. for A549^ACE2/TMPRSS2^ cells, and 48h for HAE cultures, as well as in serum collected from 11 COVID-19 patients and 10 healthy control subjects. LLT1 concentration was calculated on the basis of standard curve, and the results were shown in ng/ml.

### LLT1 treatment and CD161 blocking

NK cells were treated with LLT1 protein (Recombinant Human OCIL/CLEC2d Fc Chimera Protein, CF, R&D Systems, Minneapolis, MN, USA) at 5 µg/ml for 2h or left untreated. Next, NK cells were collected for CD161 surface flow cytometry analysis or were stimulated with a 1×cocktail of PMA and ionomycin (Cell Stimulation Cocktail, eBioscience) for 18h and then added to A549^ACE2/TMPRSS2^ cells 24h p.i. with the SARS-CoV-2 or, in the presence of 1×cocktail of Brefeldin A and Monensin (Protein Transport Inhibitor Cocktail, eBioscience), for intracellular analysis of intracellular proteins by flow cytometry, as described above.

CD161 receptor blocking on NK cells was performed by addition of 10 mg/ml of purified CD161-blocking mAb (HP-3G10, BioLegend) or isotype control IgG1 (BioLegend) followed by 2h incubation at 37°C under 5% CO_2_. Then, LLT1 protein solution at 5 µg/ml for 2h was added. Then, cytotoxicity assay or degranulation assay was performed, ot the cells were stimulated with a 1×cocktail of PMA and ionomycin (Cell Stimulation Cocktail, eBioscience) for 18h in the presence of 1×cocktail of Brefeldin A and Monensin (Protein Transport Inhibitor Cocktail, eBioscience), for intracellular analysis of intracellular proteins by flow cytometry, as described above.

### Statistical analysis

Statistical analysis was performed using GraphPad Prism version 9 (GraphPad Software Inc., San Diego, CA, USA). Where applicable, data were analyzed using t test, paired t-test or one sample t-test or Mann-Whitney test. The mean ± standard error of the mean (SEM) or median with interquartile range (IQR) was shown. The P values <0.05 were considered significant.

## Results

### SARS-CoV-2 infection results in downregulation of CD161 expression on NK cells and impairment of their function

**Figure 1:**
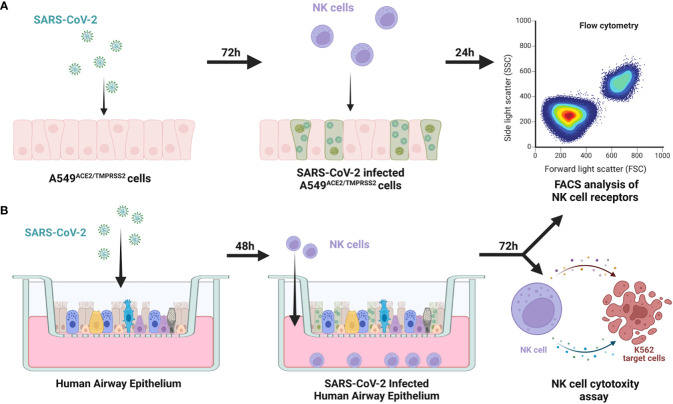
Scheme of the experimental setup. NK cells were added to 72h prior SARS-CoV-2 infected A549^ACE2/TMPRSS2^ cells, co-cultured for 24h and FACS analysis of expression of NK cell receptors was performed **(A)** NK cells were added to 48h prior SARS-CoV-2 infected HAE, co-cultured for 72h and FACS analysis of expression of NK cell receptors and cytotoxic assay against K562 cells were performed **(B)**.

First, we verified if SARS-CoV-2 infection of epithelial cells may influence the phenotype and function of circulating NK cells. For this purpose, we settled a co-culture of freshly isolated human NK cells with SARS-CoV-2 infected human epithelial A549^ACE2/TMPRSS2^ cell line, enabling direct contact of cells or using an *ex vivo* HAE model constructed with fully differentiated primary cells that mimic the physiological organization of airway tissue. NK cells were isolated by magnetic sorting from lymphocyte population isolated from healthy blood donors. NK cells were determined as CD3^-^/CD14^-^/CD16^+^CD56^+^ lymphocytes and their purity (>98%) was determined by flow cytometry (**Supplementary Figure S1**). The experimental setup was presented in **Figure 1**.

**Figure 2:**
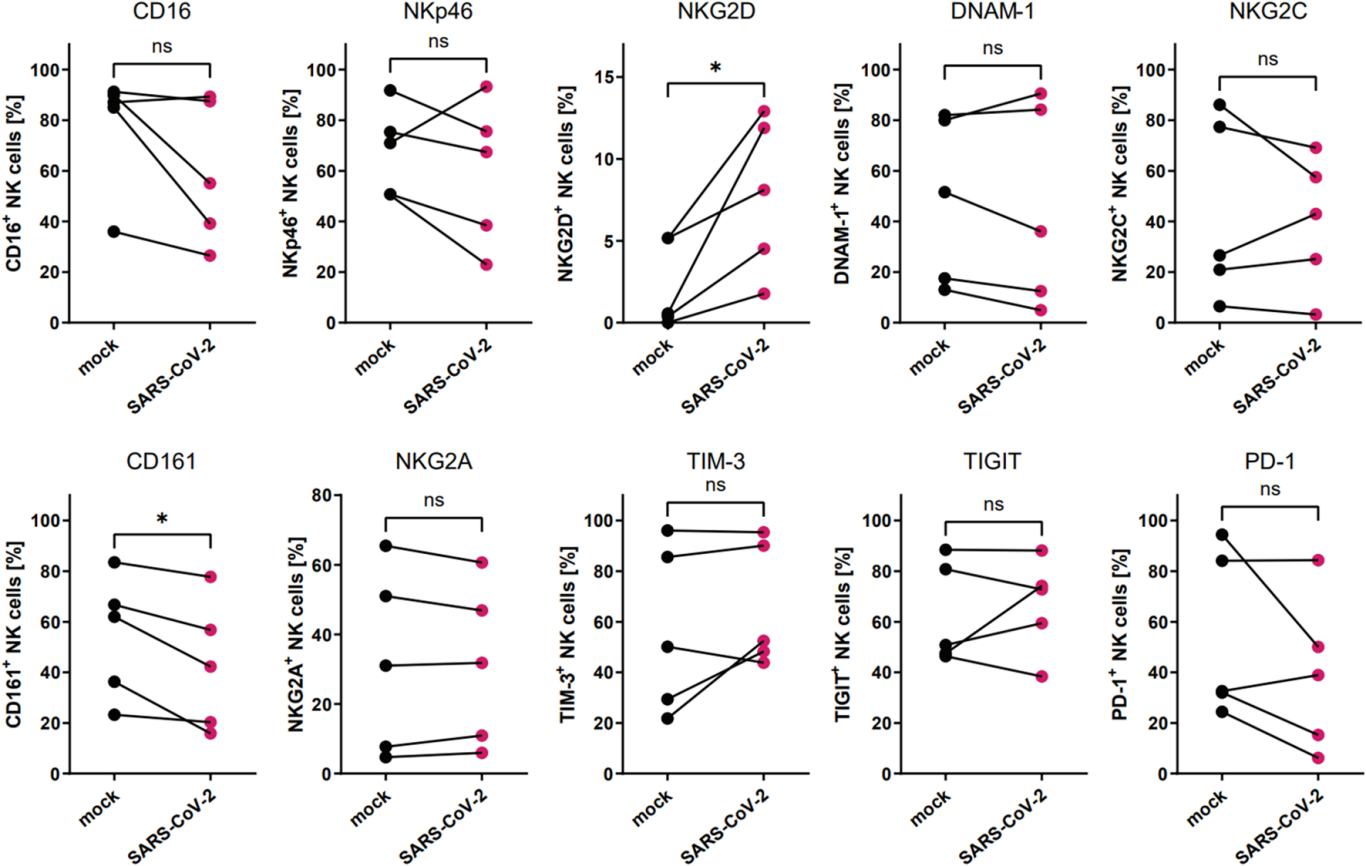
SARS-CoV-2 infection alters the proportion of NKG2D^+^ and CD161^+^ NK cells due to direct contact of NK cells with virus-infected A549^ACE2/TMPRSS2^ cells. Flow cytometry analysis of the proportion of NK cells expressing surface receptors CD16, NKp46, NKG2D, DNAM-1, NKG2C, CD161, NKG2A, TIM-3, TIGIT, and PD-1 in NK cells co-cultured with SARS-CoV-2 and mock-infected A549^ACE2/TMPRSS2^ cells. Data were obtained from five independent experiments, using NK cells isolated from five different healthy blood donors. Data were analyzed using paired t test. Asterisks mark significant differences: *p<0.05, ns, not significant.

We analyzed if the direct contact between the NK cells and SARS-CoV-2 infected cells alters the expression of surface NK cell receptors (CD16, NKG2D, NKp46, DNAM-1, NKG2C, CD161, NKG2A, TIM-3, TIGIT, and PD-1). Human epithelial A549^ACE2/TMPRSS2^ cell were infected with SARS-CoV-2 with TCID_50_ of 1600/ml or mock-infected, and after 72h of culture and NK cells were added for 24h, after which NK cell phenotype was analyzed. Viral infection was confirmed in the cell culture supernatants collected straight before the addition of NK cells, by qPCR (**Supplementary Figure S2A**). The flow cytometry analysis of NK cell receptor expression revealed a significant increase in the percentage of NK cells expressing NKG2D (7.8 ± 2.1% vs. 2.3 ± 1.2%, p=0.0332) and CD161 (42.6 ± 11.5% vs. 54.4 vs. 10.9%, p=0.0299) when compared virus- and mock-infected cells, respectively (**Figure 2**; **Supplementary Figure S3**; **Supplementary Figure S4**). At the same time, the expression levels (MFI) of CD161 and CD16 were also significantly decreased on NK cells co-cultured with virus-infected cells (**Supplementary Figure S6A**).

**Figure 3:**
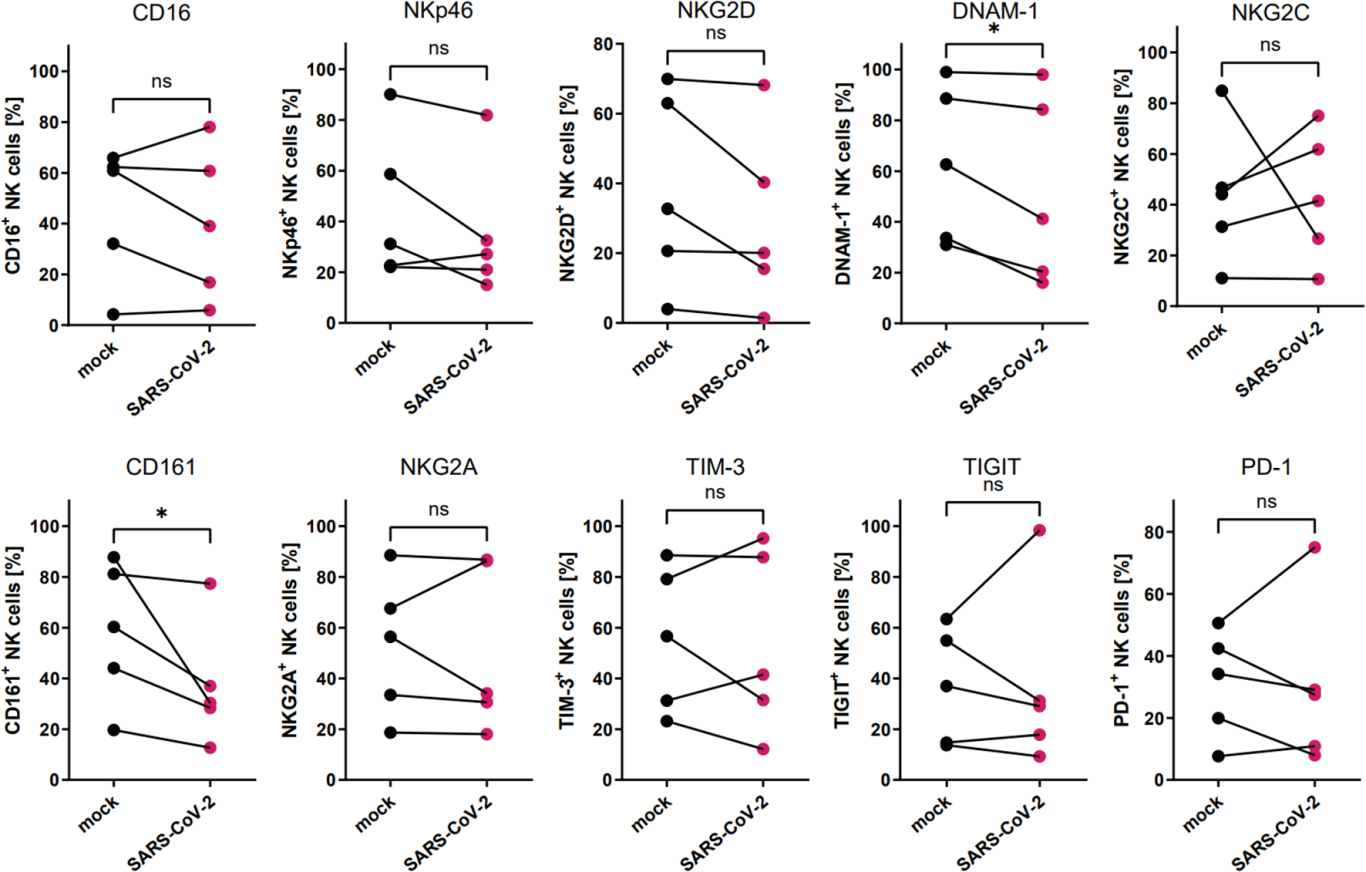
SARS-CoV-2 infection downregulates the proportion of DNAM-1^+^ and CD161^+^ NK cells due to NK cell co-culture with virus-infected HAE. Flow cytometry analysis of the proportion of NK cells expressing surface receptors CD16, NKp46, NKG2D, DNAM-1, NKG2C, CD161, NKG2A, TIM-3, TIGIT, and PD-1, in NK cells co-cultured with SARS-CoV-2 and mock-infected HAE. Data were obtained from five independent experiments, using NK cells isolated from five different healthy blood donors. Data were analyzed using paired t test. Asterisks mark significant differences: *p<0.05, ns, not significant.

**Figure 4:**
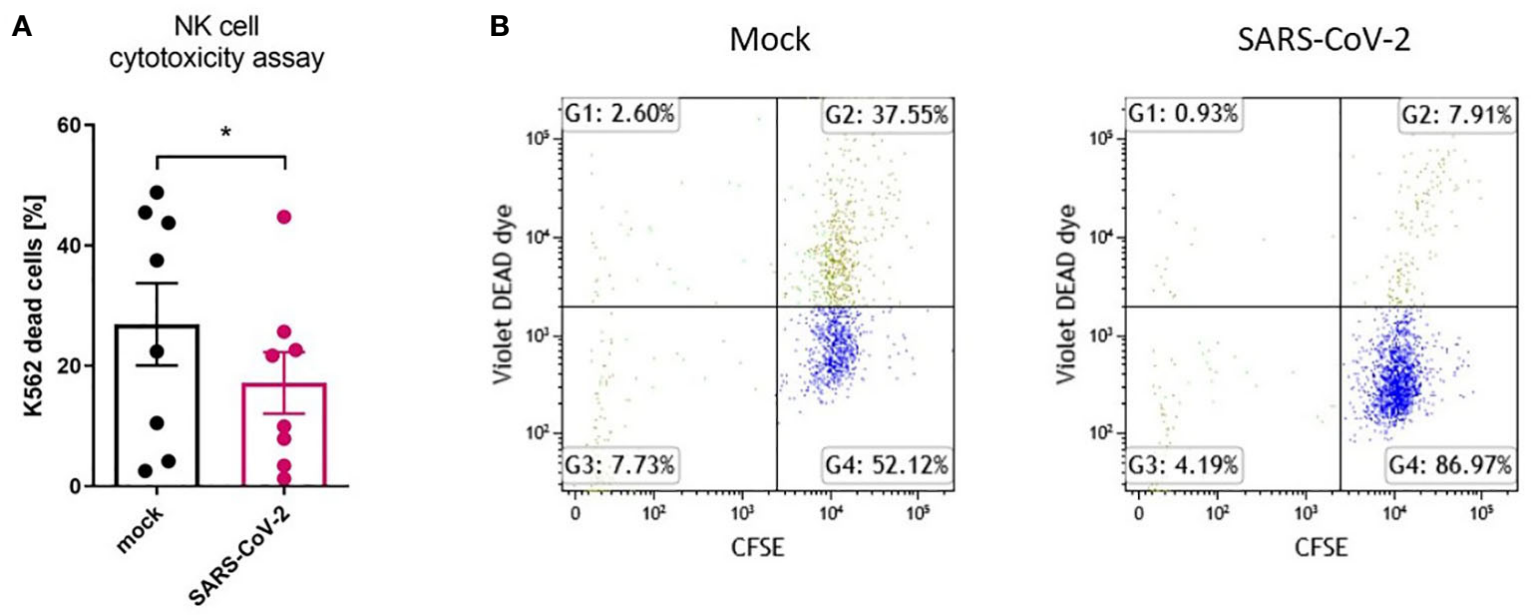
SARS-CoV-2 infection downregulates NK cell cytotoxicity after NK cell co-culture with infected HAE. NK cell cytotoxicity assay against K562 cells. Data were obtained from four independent experiments; each experiment was performed in duplicate. Data were analyzed using t test and mean ± SEM is shown **(A)**. Flow cytometry representative result. NK cells were co-cultured with CFSE-labelled K562 cells, in a ratio 5:1. Dead cells are DEAD Dye positive **(B)**. Asterisks mark significant differences: *p<0.05.

To analyze the influence of the SARS-CoV-2 infection microenvironment on NK cells in a 3D *ex vivo* model, we positioned the NK cells in the basolateral media of HAE that was SARS-CoV-2 or mock infected 48 h prior to co-culture settlement and co-cultured for another 72 h. Viral infection was confirmed in the cell culture supernatants collected straight before the addition of NK cells, by qPCR (**Supplementary Figure S2B**). Then, the flow cytometry analysis of the expression of NK cell receptors was performed and revealed significant downregulation in percentages of DNAM-1 (63.0 ± 13.9% vs. 52.0 ± 16.7%, p=0.0469) and CD161 (58.6 ± 12.4 vs. 37.2 ± 10.8%, p=0.0381) positive NK cells co-cultured with virus-infected HAE when compared to mock-infected cultures (**Figure 3**; **Supplementary Figure S3**; **Supplementary Figure S5**). However, MFI of none of the analyzed receptors on NK cells co-cultured with virus-infected HAE reached statistical significance (**Supplementary Figure S6B**). What is more, a co-culture of NK cells with SARS-CoV-2 infected HAE significantly impaired NK cells cytotoxicity level against K562 cells (**Figure 4**) compared to the co-culture with mock infected HAE (26.9 ± 6.8% vs. 17.2 ± 5.1%, p=0.0367).

These data indicate that the SARS-CoV-2 infection of epithelial cells selectively affects the proportion of CD161^+^ NK cells, and this phenomenon does not depend on direct contact between the NK and infected cells. Moreover, SARS-CoV-2 infection of primary epithelial cells reduces the cytotoxicity capacity of NK cells.

### CD161^+^ NK cells more effectively limit SARS-CoV-2 infection

**Figure 5:**
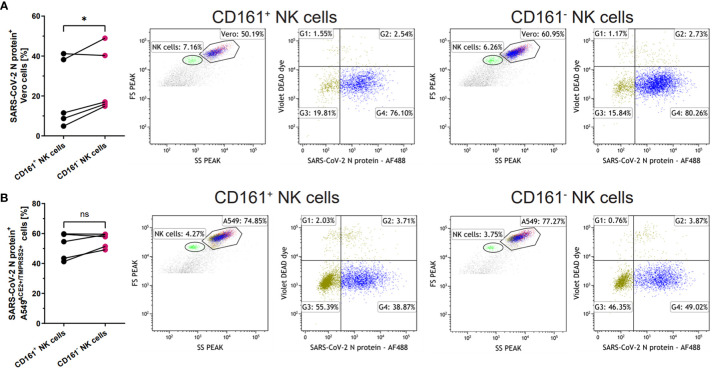
CD161^+^ NK cells more effectively limit SARS-CoV-2 infection. Sorted CD161^+^ and CD161^-^ NK cells were added onto SARS-CoV-2 infected Vero **(A)** or A549^ACE2/TMPRSS2^ **(B)** cells 24h p.i. Infected cells were analyzed based on the intracellular expression of SARS-CoV-2 N protein, while live Vero and A549^ACE2/TMPRSS2^ cells were analyzed on FSC/SSC scatter and DEAD Dye staining. The percentage of live SARS-CoV-2 N protein^+^ cells were calculated on the basis of the percentage in G4 quadrant and the percentage of Vero **(A)** or A549^ACE2/TMPRSS2^ **(B)** cells. The flow cytometry plots shows representative results. Data were obtained from five independent experiments. Data were analyzed using paired t test and means ± SEM are shown. Asterisks mark significant differences: *p<0.05, ns, not significant.

Next, we focused on the CD161 receptor, as its levels on the NK cells were decreased in all experimental setups after contact with SARS-CoV-2 infected cells. Due to the conflicting literature on the role of this receptor, either as activating (21) or inhibitory (10), we verified the CD161-positive and CD161-negative NK cells ability to modulate the infection. Sorted CD161^+^ and CD161^-^ NK cells (**Supplementary Figure S7**) were added, in the same proportions, to Vero or A549^ACE/TMPRSS2^ cells infected 24h earlier with the SARS-CoV-2 virus. The intracellular expression of SARS-CoV-2 N protein was determined by flow cytometry 24h later (**Supplementary Figure S8** and **Figure 5**). We observed a significant reduction in the number of virus-infected live Vero cells after the co-culture with CD161^+^ NK cells when compared to CD161^-^ cells (20.1 ± 7.8% vs. 27.4 ± 7.2%, p=0.0359, respectively) (**Figure 5A**). In the case of A549^ACE2/TMPRSS2^ cells, a similar pattern is visible (**Figure 5B**), but the difference did not reach statistical significance.

### Upregulation of CD161 ligand LLT1/CLEC2D in SARS-CoV-2 infected cells

**Figure 6:**
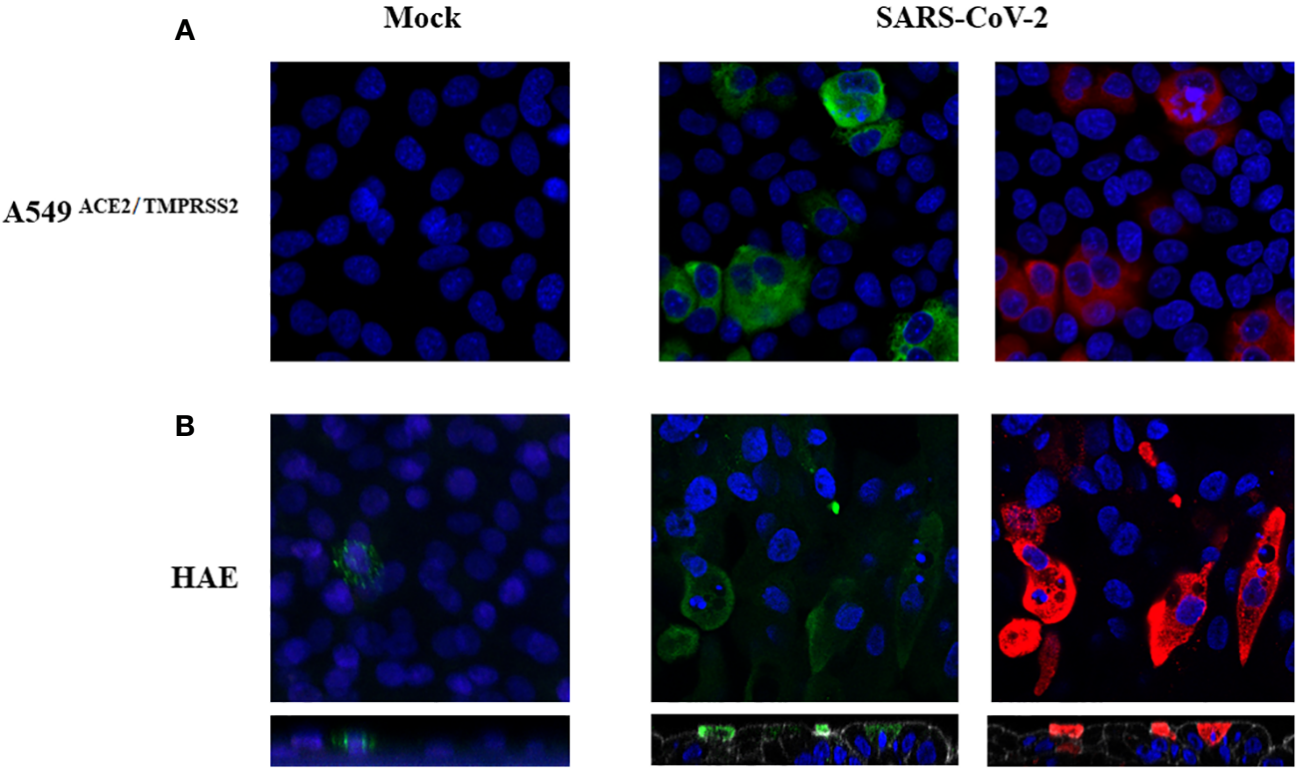
SARS-CoV-2 infection upregulates LLT1 expression on infected epithelial cells. Confocal microscopy images of A549^ACE2/TMPRSS2^ cells **(A)** and HAE **(B)**, mock or virus-infected. Images were obtained at 48h p.i. **(A)**, or 104h p.i. **(B)**. SARS-CoV-2 N protein is shown in red, LLT1 in green, and nuclei in blue. The bottom panels show an orthogonal view of cells (XZ projection) **(B)**.

Since the CD161 ligand, LLT1 was previously shown to downregulate CD161 surface NK cells expression (24), we verified its level on SARS-CoV-2 infected cells. We immunostained virus or mock-infected A549^ACE2/TMPRSS2^ cells (48h p.i) and HAE cultures (96h p.i) to visualize LLT1 expression. Confocal images show upregulated expression of LLT1 in SARS-CoV-2 infected A549^ACE2/TMPRSS2^ cells (**Figure 6A**) and HAE cultures (**Figure 6B**) compared to mock-infected controls. Furthermore, LLT1 expression was most pronounced in cells expressing high quantities of viral proteins. We then wondered, if LLT1 protein is being shedded from infected cells and might be detected in the cell culture supernatant. Thus, we analyzed LLT1 concentration in SARS-CoV-2 infected A549^ACE2/TMPRSS2^ cell supernatants, HAE basolateral medium and mock-infected controls, using ELISA. The samples were collected straight before the addition of NK cells, that is 48h for HAE cultures and 72h p.i. for A549 cells. As a result, we detected 237 +/- 150 ng/ml of LLT1 protein in SARS-CoV-2 A549^ACE2/TMPRSS2^ cell supernatants and 225 +/- 135 ng/ml in SARS-CoV-2 infected HAE basolateral medium, while LLT1 protein level in mock-treated control cultures was below the detection level (OD was lower that OD of blank). Thus, results of confocal imaging of surface cellular LLT1 expression and ELISA results suggest that LLT1 protein is not only expressed on SARS-CoV-2 infected epithelium, but also is cleaved or secreted from the cell membrane and may influence NK cell disregarding their direct contact with infected cell.

Additionally, we conducted a pilot study in which we analyzed LLT1 protein concentration in the serum of 11 COVID-19 patients admitted to the hospital due to pneumonia and 10 healthy control subjects. We detected the presence of LLT1 in the serum of 8 patients and 4 healthy controls, with mean concentrations of 0 (IQR 0.0; 307.6) ng/ml and 173.6 (IQR 0.0; 495.3) ng/ml (median with IQR), respectively (**Supplementary Figure S9**).

### LLT1 downregulates NK cell CD161 expression and impairs NK cell functions, and their ability to control SARS-CoV-2 infection

**Figure 7:**
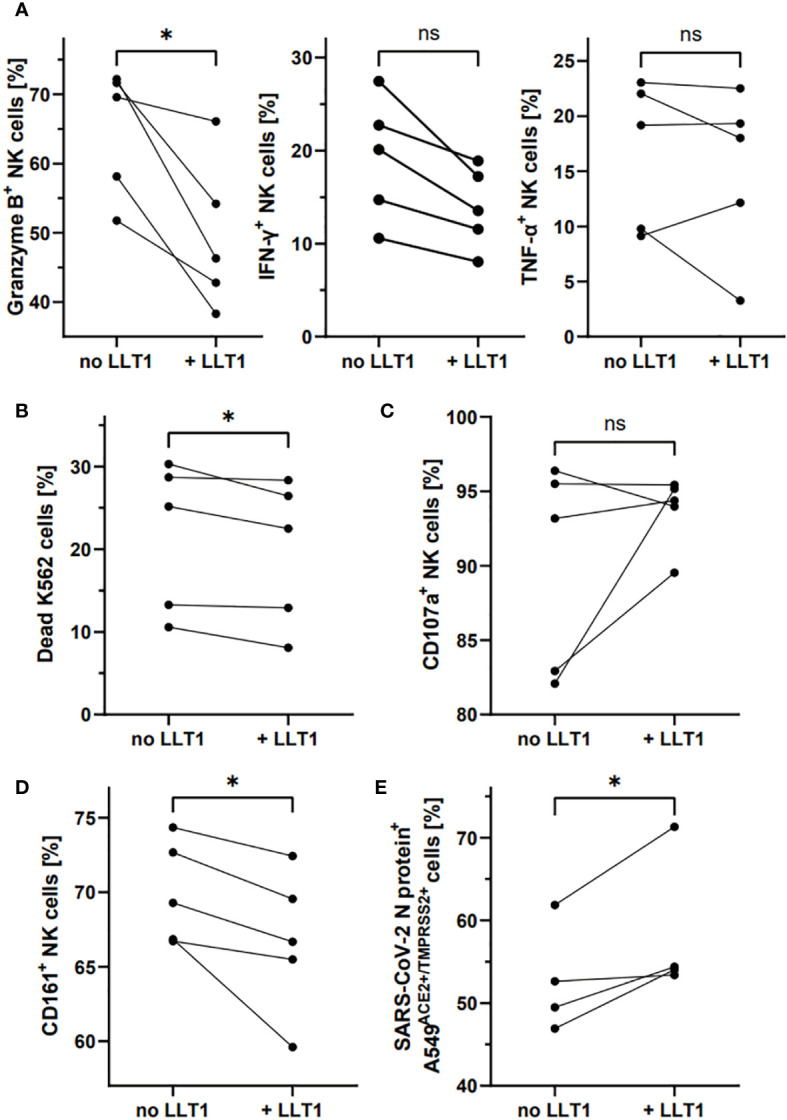
LLT1 treatment downregulates CD161^+^ NK cell proportion and impairs NK cell functions and ability to control SARS-CoV-2 infection. NK cells treated for 2h with LLT1 protein, followed by 18h stimulation with PMA/ionomycin, were analyzed for intracellular expression of granzyme B, IFN-γ, and TNF-α **(A)**. NK cells were treated for 2h with LLT1 protein and their cytotoxicity capacity was analyzed against K562 cells **(B)** or their degranulation level measured as CD107a surface expression **(B)**. The proportion of CD161^+^ NK cells was measured after 2h treatment with soluble LLT1 protein **(C)**. SARS-CoV-2 infected cells were analyzed based on the intracellular expression of SARS-CoV-2 N protein **(D)**. Data were obtained from five independent experiments and analyzed using paired t test. Asterisks mark significant differences: *p<0.05, ns, not significant.

Finally, we tested whether LLT1 protein can affect the phenotype and function of NK cells, as well as their ability to control SARS-CoV-2 infection (**Figure 7**). For this purpose, we treated freshly isolated NK cells with soluble LLT1 protein (5 µg/ml). Moreover, NK cells treatment with LLT1 for 2h, followed by 18h stimulation with PMA/ionomycin, resulted in a significant decrease of the proportion of NK cells expressing intracellularly granzyme B (46.3 ± 4.6% vs. 67.3 ± 4.6%, p<0.001) compared to the LLT1-untreated cells while IFN-γ and TNF-α expression was not altered (**Figure 7A**, **Supplementary figure S10**). LLT1 treatment was also observed to downregulate NK cell cytotoxic capacity toward target K562 cells (19.6 ± 3.9% vs. 21.6 ± 4.1%, p=0.0472, respectively) (**Figure 7B**), yet does not change CD107a degranulation marker expression on NK cells (93.7 ± 1.1 vs. 90.0 ± 3.1, p=0.2556) (**Figure 7C**). Moreover, 2h of LLT1 treatment resulted in a significant downregulation of CD161-expressing NK cells (66.87 ± 2.2% vs. 70.0 ± 1.5%, p=0.0375, when LLT1-treated to LLT1-untreated NK cells were compared, respectively) (**Figure 7D**). Finally, LLT-1 treatment of NK cells, followed by 18h stimulation with PMA and ionomycin, significantly reduced the ability of NK cells to control SARS-CoV-2 infection in A549^ACE2/TMPRSS2^ cells. The latter, infected 24h prior to the addition of stimulated NK cells, were analyzed for intracellular SARS-CoV2 N protein expression after 24h co-culture. The results showed significantly higher levels of the N protein in cells co-cultured with LLT1-treated NK cells compared to untreated NK cells (58.3 ± 4.4% vs. 52.8 ± 3.3%, p=0,0473, respectively) (**Figure 7E**).

**Figure 8:**
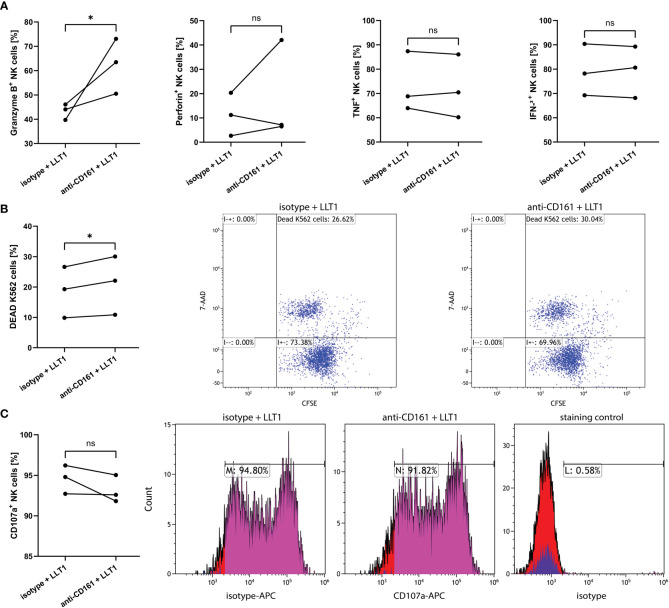
CD161 blocking mAb pre-treatment of NK cells upregulates the proportion of granzyme B expressing NK cells and induce their cytotoxicity capacity, yet not the degranulation level. NK cells pre-treated with anti-CD161 blocking mAb (clone HP-3G10) or isotype control, and then treated for 2h with LLT1 protein, followed by 18h stimulation with PMA/ionomycin, were analyzed for intracellular expression of granzyme B, perforin, IFN-γ, and TNF-α **(A)**. NK cells were pre-treated with anti-CD161 blocking mAb (clone HP-3G10) or isotype control, and then treated for 2h with LLT1 protein and their cytotoxicity capacity was analyzed against K562 cells **(B)** or their degranulation level measured as CD107a surface expression **(C)**. FACS plots show representative results of cytotoxicity assay against CFSE-labelled K562 cells **(B)** or CD107a degranulation assay **(C)**. Data were obtained from three independent experiments and analyzed using paired t test. Asterisks mark significant differences: *p<0.05, ns, not significant.

To support these data, we treated NK cells with anti-CD161 blocking mAb or isotype control (10), followed by LLT1 treatment, and analyzed intracellular production of NK cell cytolytic proteins and cytokines, NK cell cytotoxic capacity and degranulation level. CD161 blockage resulted in significant upregulation of granzyme B^+^ cells, when compared to isotype treated NK cells (62.4 ± 6.5% vs. 43.3 ± 1.9%, p=0.0484, respectively), yet the expression of perforin, IFN-γ and TNF-α was not influenced (**Figure 8A**). The pre-treatment with anti-CD161 mAb significantly upregulated NK cell cytotoxic abilities toward K562 cells (21.0 ± 5.6% vs. 18.6 + 4.8%, p=0.0103) (**Figure 8B**), yet did not change the percentage of CD107a^+^ NK cells (93.2 ± 1.0% vs. 94.6 ± 1.0%, p=0,2287) (**Figure 8C**).

**Figure 9:**
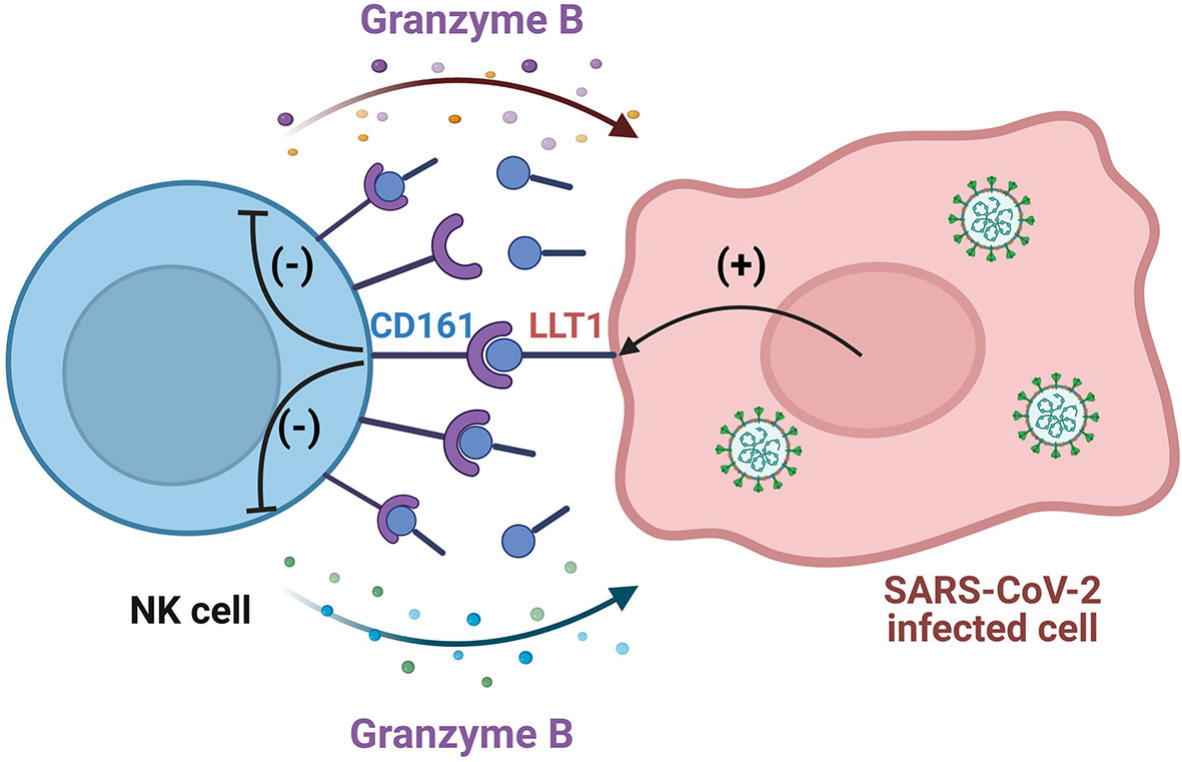
Scheme of SARS-CoV-2 mediated NK cell impairment by the activation of the LLT1-CD161 axis. LLT1 expression is upregulated in SARS-CoV-2 infected epithelial cells. LLT1 acts as a ligand for the NK cell CD161 receptor, which, in turn, inhibits granzyme B production by NK cells, inhibiting NK cell-mediated antiviral functions, their cytotoxicity capacity, yet not the degranulation level. Created with BioRender.com.

These data indicate that LLT1 treatment of NK cells results in the downregulation of NK cell CD161 surface expression, hampers NK cell cytotoxic capacities associated with granzyme B production, yet not the degranulation level, and promotes SARS-CoV-2 infection of epithelial cells (**Figure 9**).

## Discussion

The role of NK cells in controlling human coronaviruses has been understudied for years. Numerous observations of NK cell imbalance and aberrated function in COVID-19 patients (13, 14, 25–28) raised the justified question whether these frontline of the immune system may predetermine the course of COVID-19 disease. Various mechanisms were proposed, ranging from abrogated NK cell activation *via* activating receptors (17) to enhanced NK cell inhibitory receptors stimulation (16). Here, we propose that SARS-CoV-2 hampers the NK cell antiviral activity *via* activation of the LLT1-CD161 axis.

The alterations of NK cell receptor expression, both activating and inhibitory, have been previously shown in COVID-19 patients. Witkowski M. et al. (17) suggested the role of activating NK cell receptors in the course of SARS-CoV-2 infection. In this study, a blockage of single, activating NK cell receptors did not impair virus control, whereas simultaneous blockade of all three natural cytotoxicity receptors (NKp30, NKp44 and NKp46) or of 2B4, NKG2D and DNAM-1 led to a significant increase in SARS-CoV-2 replication in Calu-3 cells. The Authors thus concluded that NK-cell-mediated control of SARS-CoV-2 replication in infected target cells requires redundant recognition by activating NK cell receptors, while this process was impaired in infected cells treated with NK cells isolated from patients admitted to hospital with COVID-19. In line with *Witkowski M. et al.* (17) results, noticed a significant downregulation of DNAM-1^+^ NK cells after their co-culture with SARS-CoV-2 infected HAE. However, it is worth to emphasize, that expression of activating receptors can be decreased upon ligand interactions or upon surface shedding by metalloproteases, as was already shown in case of CD16. The shedding of CD16 was suggested to positively impact immune responses, as CD16 shedding also increased NK cell motility and facilitated detachment of NK cells from target cells (29). Indeed, in our study we observed a downregulated CD16 expression level on NK cells co-cultured with SARS-CoV-2 infected A549^ACE2/TMPRSS2^ cells. Moreover, a co-culture of NK cells with SARS-CoV-2 infected A549^ACE2/TMPRSS2^ cells induced an upregulation of NKG2D^+^ NK cells, and the role of NKG2D receptor in an induction of severe lung inflammation was recently suggested (30).

In our study, NK cell contact with SARS-CoV-2 infected microenvironment, organized similarly to airway tissue in which SARS-CoV-2 infection begins, might alter NK cell cytotoxic capacity, which might be associated with the exhaustion state of NK cells. However, we did not observed significantly upregulated expression of exhaustion markers/inhibitory NK cell receptors (PD-1, TIM-3, TIGIT), either as a percentage of receptor-expressing NK cells, or the expression levels determined as MFI. There is increasing evidence suggests that inhibitory checkpoint receptors on NK cells might contribute to the dysfunctional status of COVID-19-associated NK cells. For example, *Demaria et al.* observed higher levels of NKG2A, PD-1, and CD39 in COVID-19-associated NK cells from the peripheral blood and bronchoalveolar lavage fluid (15), while *Krämer et al.* detected increased TIGIT expression on NK cells in some COVID-19 patients (14).

Previous studies showed contradictory data, suggesting that CD161 is either activating (21, 31) or inhibitory (10, 20) NK cell receptor. It was demonstrated that CD161 marks NK cells that have retained the ability to respond to innate cytokines during their differentiation; this marker is lost upon cytomegalovirus-induced maturation (21). On the other hand, transfection of LLT1 into NK target cells protects them from human NK cell cytotoxicity in a CD161-dependent manner (22), while NK cell treatment with soluble LLT1 protein leads to inhibition of NK cell cytotoxicity towards target cells, both in our study and recently published study of Blaha et al. (32). In agreement with other reports (21) indicating that CD161 expression on NK cells marks a pro-inflammatory NK cell subset, we observed that CD161^+^ NK cells more effectively limit SARS-CoV-2 infection. CD161 expression is known to be higher on CD56^dim^CD16^bright^ NK) cell subset with higher effector response (31, 33), thus it would be possible that observed lower expression of CD161 may be rather associated with lower percentage of this specific NK cell subset. However, we did not notice any significant alterations of alterations of NK cell subsets, CD56^dim^CD16^bright^ and CD56^bright^CD16^dim^, in response to NK cell contact with the virus- or mock-infected epithelium *(data not shown)*. Our data show, though, that CD161 expression on NK cells coincides with high expression of activating receptors, such as NKp46, NKG2D, and DNAM-1 *(data not shown)*. However, considering the inhibitory role of the CD161 receptor on NK cells, we cannot exclude that high expression of this receptor on the most potent pro-inflammatory NK cell subset serves as a safety valve, protecting from excessive NK cell-mediated inflammation.

It is also worth mentioning, that anti-CD161 mAb clone HP-3G10, commonly used for flow cytometry analysis of CD161 expression or FACS sorting of CD161^+^ and CD161^-^ cells, was shown to act as a CD161 receptor blocking mAb (10). In our study, anti-CD161 mAb clone HP-3G10 reduced LLT1-mediated inhibition of NK cell functions, suggesting the need of application of different clone anti-CD161 mAb for LLT1/CD161 interactions studies.

Upregulation of LLT1 expression was previously linked with immune evasion or modulation of the immune response. Airway epithelial cells upregulated LLT1 expression following respiratory syncytial virus (RSV) infection, yet, in contrast to our observations, it was associated with an increase in proinflammatory cytokine (i.e., type I interferons, IL-1β and TNF-α) production (34). LLT1 expression is also upregulated in non-infectious human diseases, associated with immune response dysregulation. The expression of LLT1 on cancer cells, such as triple-negative breast cancer cells and prostate cancer cells, inhibits the NK cell response (19, 20). Additionally soluble LLT1 protein was found in serum isolated from rheumatoid arthritis patients (35), supporting our observations that the LLT1 mode of action is not limited to direct contact between NK cells and LLT1-expressing cells. Thus, we might postulate that LLT1 secretion from SARS-CoV-2 infected tissue could be partially associated with NK cell inhibition observed in COVID-19 patients. Our results suggest that the LLT1 protein is present in the circulation and might influence circulating NK cell response during infection, which has previously been shown to be downregulated in COVID-19 patients (13, 14). Further studies with larger sample sizes may be necessary to better understand the potential role of LLT1 in the circulation and its impact on NK cell responses during SARS-CoV-2 infection. What is more, LLT1-coding gene, CLEC2D, was shown to be one of 10 hub differentially expressed genes (DEGs) between COVID-19 ARDS and control group patients in the bioinformatics analysis of RNA-sequencing dataset of COVID-19 ARDS, obtained from GSE163426, Gene Expression Omnibus (GEO) database (36). These data indicate that LLT1/CLEC2D might be an important player in SARS-CoV-2 mediated ARDS development in COVID-19 patients and may support our findings of LLT1 role in SARS-CoV-2-mediated immune response alterations.

SARS-CoV-2 infection of epithelial cells was previously shown to cause NK cell exhaustion, *via* increased modulation of the inhibitory receptor NKG2A/CD94 on NK cells by SARS-CoV-2 spike protein S1 subunit (16). Bortolotti et al. showed that lung epithelial cell transfection with S1 subunit and their co-culture with NK cells, resulted in NK cell-reduced degranulation. This phenomenon was associated with increased expression of inhibitory receptor NKG2A and its ligand HLA-E on NK cells and epithelial cells, respectively. Further studies revealed that HLA-E of SARS-CoV-2 infected cells present a peptide encoded by Non-structural protein 13, which prevents binding of HLA-E to NKG2A, thereby rendering target cells susceptible to NK cell attack. NKG2A-expressing NK cells are particularly activated in patients with COVID-19 and proficiently limit SARS-CoV-2 replication in infected lung epithelial cells *in vitro* (37). However, in our studies, we did not observe significant aberrations in NK cell NKG2A expression, both in NK cells co-cultured with A549^ACE2/TMPRSS2^ cells and HAE cultures. What is more, we did not notice any changes in HLA-E expression on A549^ACE2/TMPRSS2^ cells *(data not shown)*. Thus, our data support the belief of the limited role of HLA-E/NKG2A axis in SARS-CoV-2 mediated NK cell exhaustion.

## Conclusions

We propose a new mechanism of SARS-CoV-2 inhibition of NK cell functions *via* activation of the LLT1-CD161 axis. LLT1 expression is upregulated in SARS-CoV-2 infected epithelial cells. LLT1 acts as a ligand of the NK cell CD161 receptor, inhibiting at least granzyme B and IFN-γ production by NK cells, hampering NK cell-mediated antiviral functions.

## Data availability statement

The raw data supporting the conclusions of this article will be made available by the authors, without undue reservation.

## Ethics statement

The studies involving human participants were reviewed and approved by the Local Bioethics Committee (78/KBL/OIL/2020) and complied with the Declaration of Helsinki and good clinical practice guidelines.. The patients/participants provided their written informed consent to participate in this study.

## Author contributions

Conceptualization: ML and KP. Data curation: ML. Data analysis: ML, MB, AS, PL and KP. Patients recruitment: AS-K, MK. Funding acquisition: ML and KP. Methodology and data acquisition: ML, MG, EB-D, AS, AG-B, NM-P and KW. Supervision: KP. Manuscript writing: ML. Manuscript review: KP, MB-K and MS. All authors contributed to the article and approved the submitted version.
