# Supplementary material for: SARS-CoV-2 infection impairs NK cell functions via activation of the LLT1-CD161 axis
Source: Front Immunol. 2023 May 23;14:1123155. doi: 10.3389/fimmu.2023.1123155 (PMC10242091; doi:10.3389/fimmu.2023.1123155)

Supplementary Figures

Supplementary Figure S1. Flow cytometry analysis of isolated NK cell population purity. NK cells were gated in the lymphocyte region on the FSC/SSC plot (A), then as the $CD3^-$ (B) and $CD14^-$ cells (C), and finally, NK cells exhibited either $CD56^{bright}CD16^{dim}$, $CD56^{bright}CD16^{bright}$ or $CD56^{dim}CD16^{bright}$ phenotype (D).

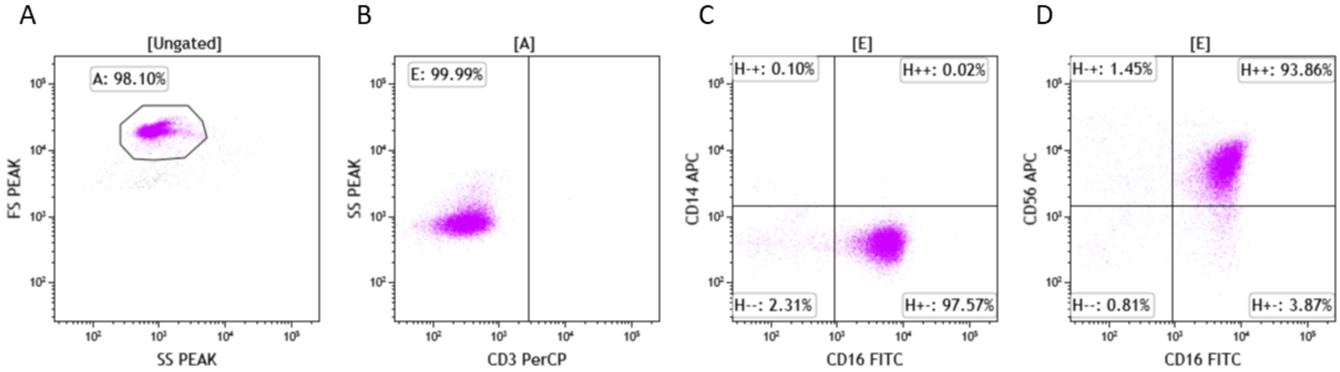

Supplementary Figure S2. Confirmation of SARS-CoV-2 infection of A549$^{ACE2/TMPRSS2}$ cells (A) and HAE (B). RT-qPCR results encompassing the number of SARS-CoV-2 copies per ml in 2 h post-infection and directly before the addition of NK cells, i.e., 72 h p.i. for A549$^{ACE2/TMPRSS2}$ cells (A), and 48h for HAE cultures (B). Data were obtained from three (A) or five (B) independent experiments, in duplicate (A) or quadruplicate (B) and median ± IQR is shown. Data were analyzed using Mann-Whitney test. Asterisks mark significant differences: ****p<0.0001, **p<0.01.

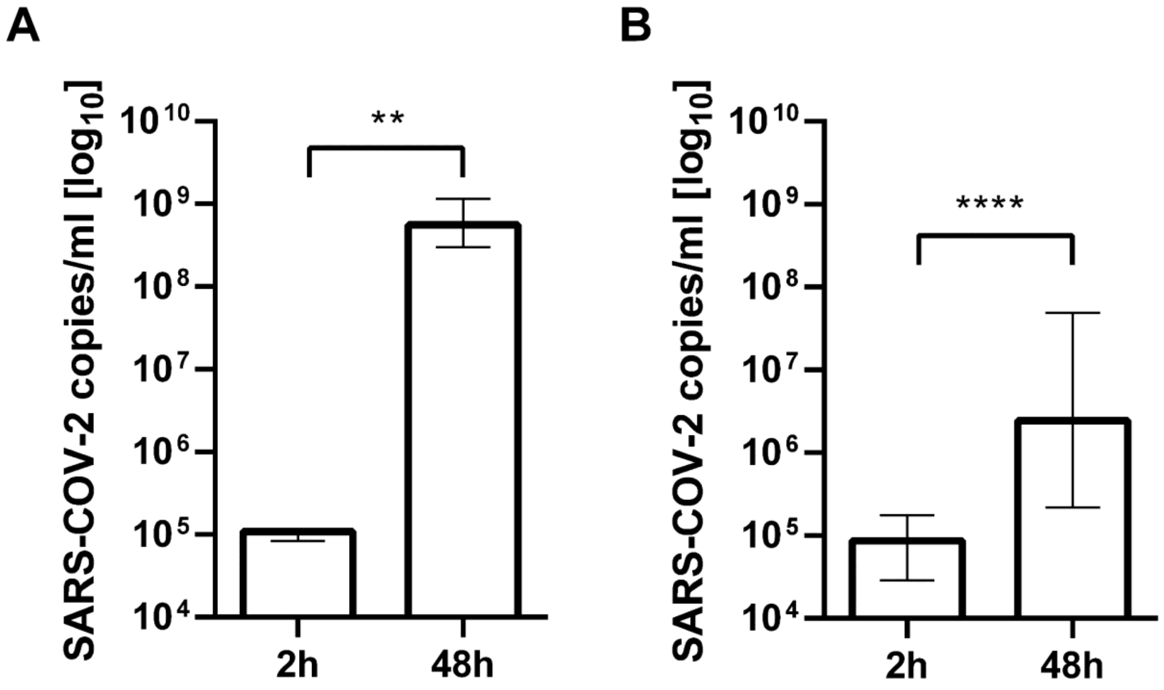

Supplementary Figure S3. Flow cytometry analysis of NK cell receptor expression. An overlayer of representative histograms of NK cell receptor expression with appropriate controls. The analysis of NKG2D (A), NKp46 (B), DNAM-1 (C), CD161 (D), NKG2A (E), NKG2C (F), PD-1 (G), TIGIT (H), TIM-3 (I). Abbreviations: FMO – fluorescence minus one, ISO – isotype control.

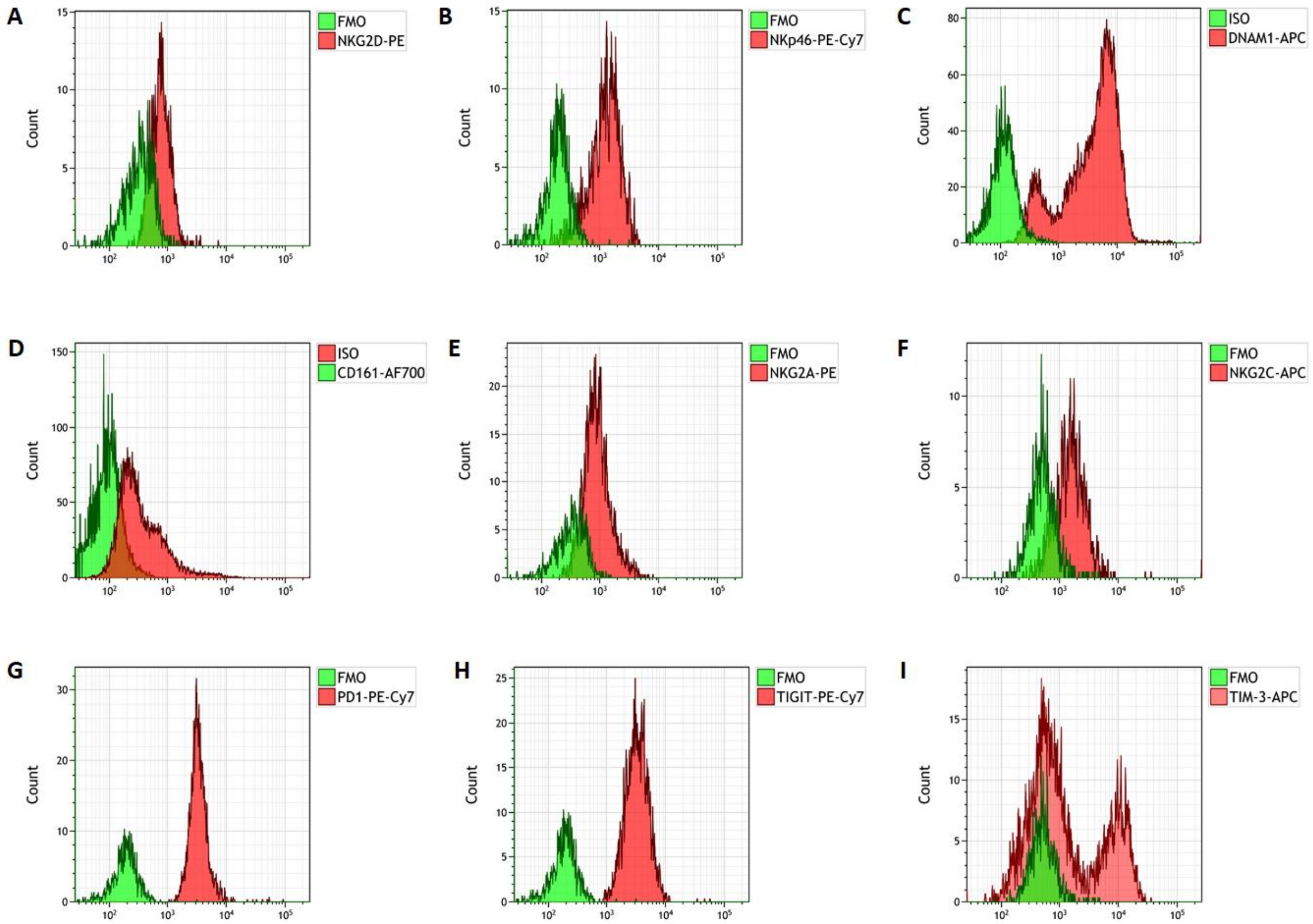

Supplementary Figure S4. Gating strategy of FACS analysis of receptor expression on NK cells co-cultured with A549$^{ACE2/TMPRSS2}$ cells. NK cells were gated in the lymphocyte region on the FSC/SSC plot, then as the CD16$^+$CD56$^+$ cells, and finally, the expression of analyzed NK cell receptor was analyzed.

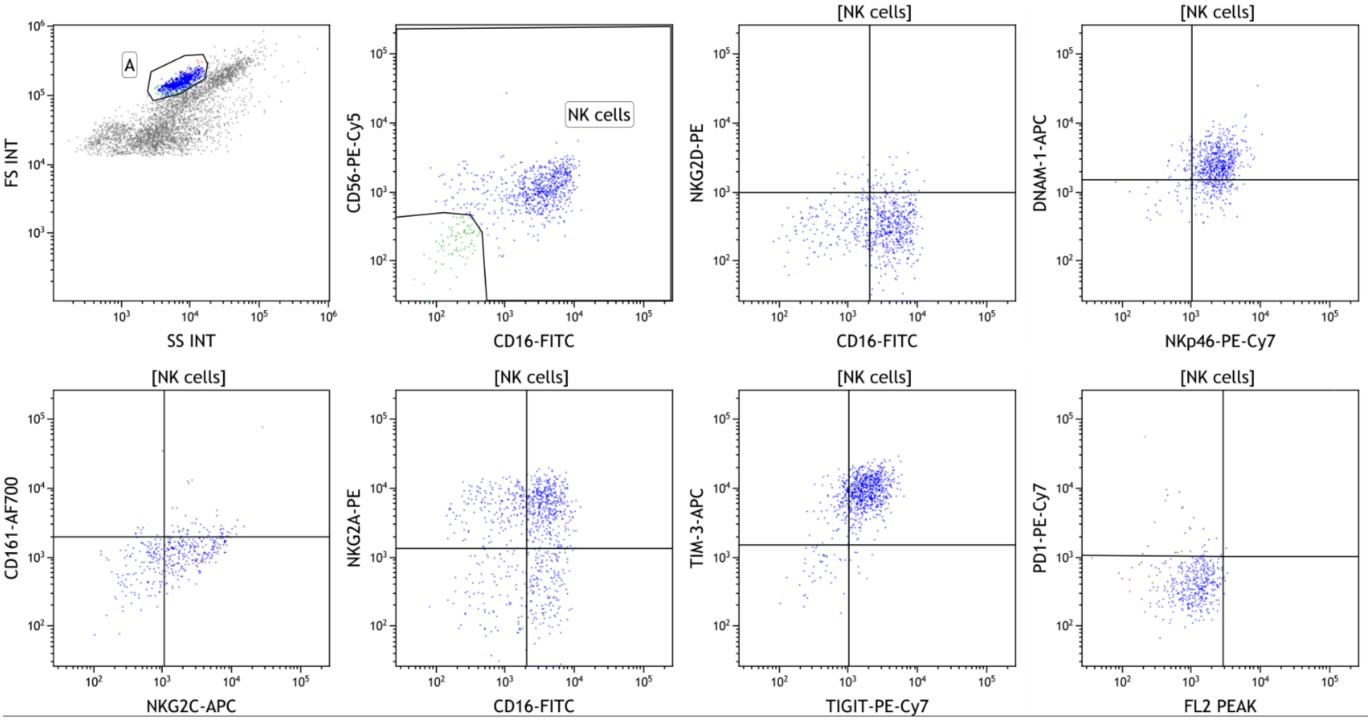

Supplementary Figure S5. Gating strategy of FACS analysis of receptor expression on NK cells co-cultured with HAE. NK cells were gated in the lymphocyte region on the FSC/SSC plot, then as the CD16^{+}CD56^{+} cells, and finally, the expression of analyzed NK cell receptor was analyzed.

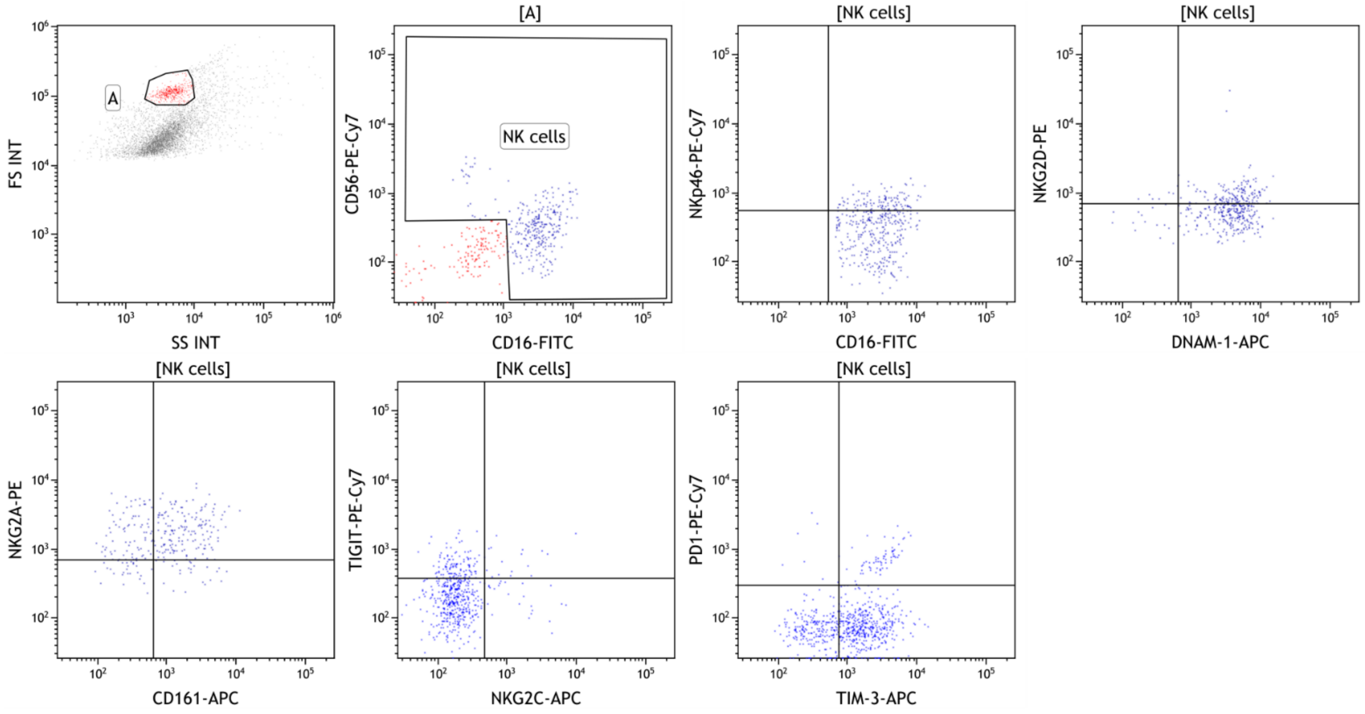

Supplementary Figure S6. SARS-CoV-2 infection significantly downregulates CD16 and CD161 expression level on NK cells co-cultured with virus-infected A549$^{ACE2/TMPRSS2}$ cells. Fold change between NK cell receptor determined as MFI, between NK cells co-cultured with SARS-CoV-2 and mock-infected A549$^{ACE2/TMPRSS2}$ cells (A) or HAE (B). Data were obtained from five independent experiments, and means ± SEM is shown. Data were analyzed using one-sample t test. Asterisks mark significant differences: *$p<0.05$, ns, not significant.

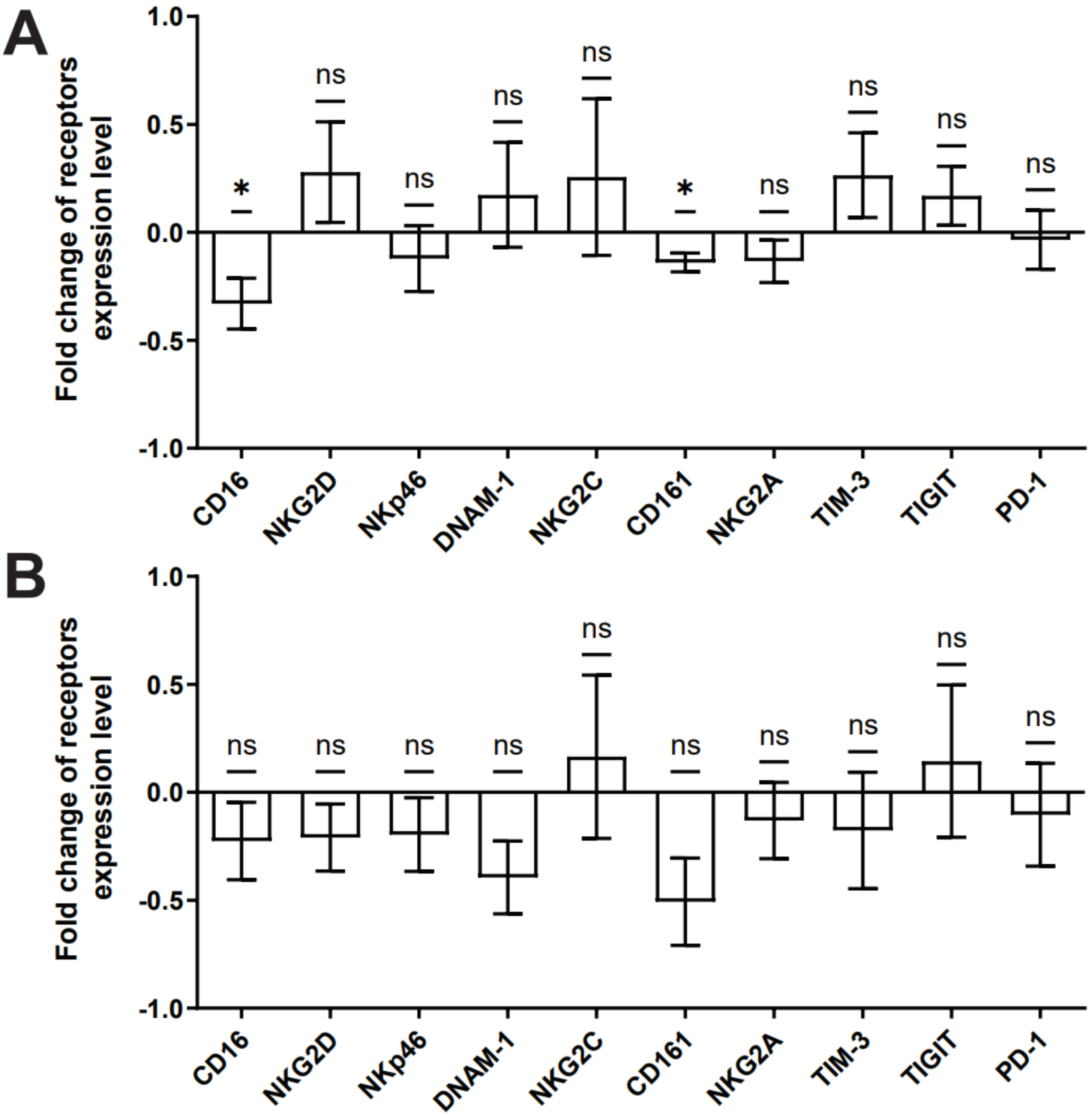

Supplementary Figure S7. CD161^{+} and CD161^{-} NK cell sorting gating strategy. NK cells were gated in the lymphocyte region on the FSC/SSC plot (A), then as the CD3^{-}(B) and CD16^{+}CD56^{+} cells (C). NK cells were then sorted on the basis of surface CD161 expression, to CD161^{+} (CD161pos) and CD161^{-} (CD161neg) populations (D).

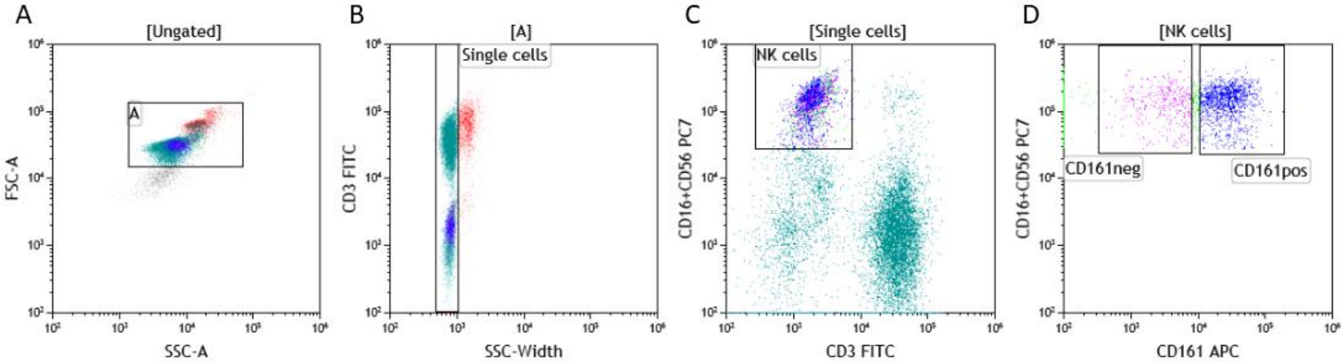

Supplementary Figure S8. SARS-CoV-2 N protein intracellular expression in virus or mock infected Vero (A) or A549$^{ACE2/TMPRSS2}$(B) cells 48h p.i. The flow cytometry plots shows representative results.

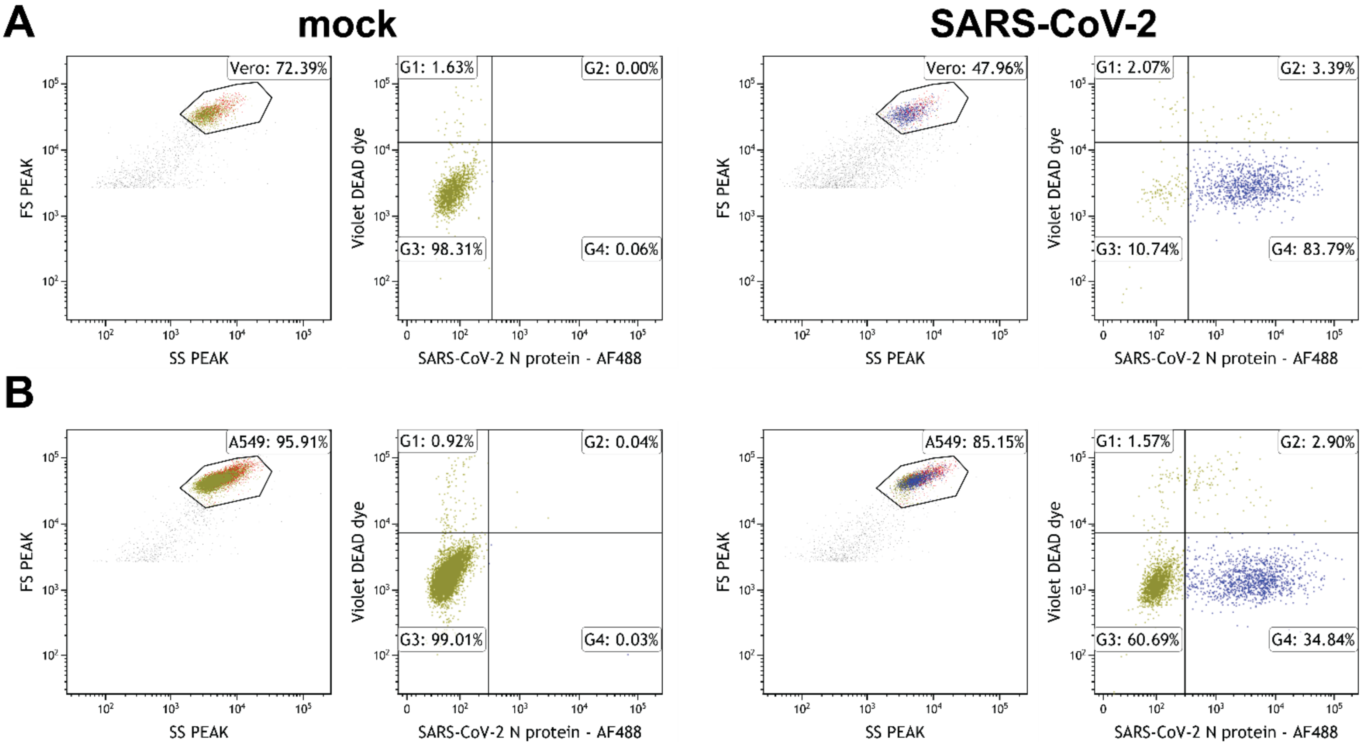

Supplementary Figure S9. LLT1 protein concentration in serum of COVID-19 patients and healthy controls. LLT1 protein levels in serum was analyzed using ELISA. The data were analyzed using Mann-Whitney test and the median ± IQR is shown. Asterisks mark significant differences: ns, not significant.

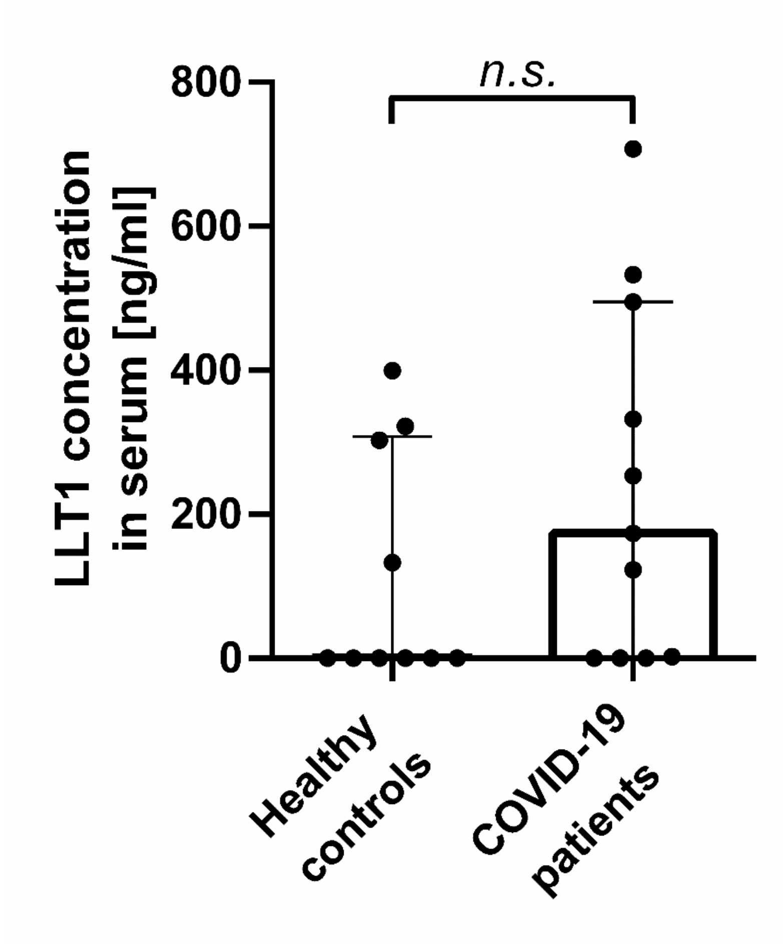

Supplementary Figure S10. Representative plots of FACS analysis of intracellular markers expression on NK cells untreated (A) or treated (B) with LLT1 protein. NK cells were gated in the lymphocyte region on the FSC/SSC plot, then the expression of granzyme B, IFN-γ and TNF-α in NK cell was analyzed.

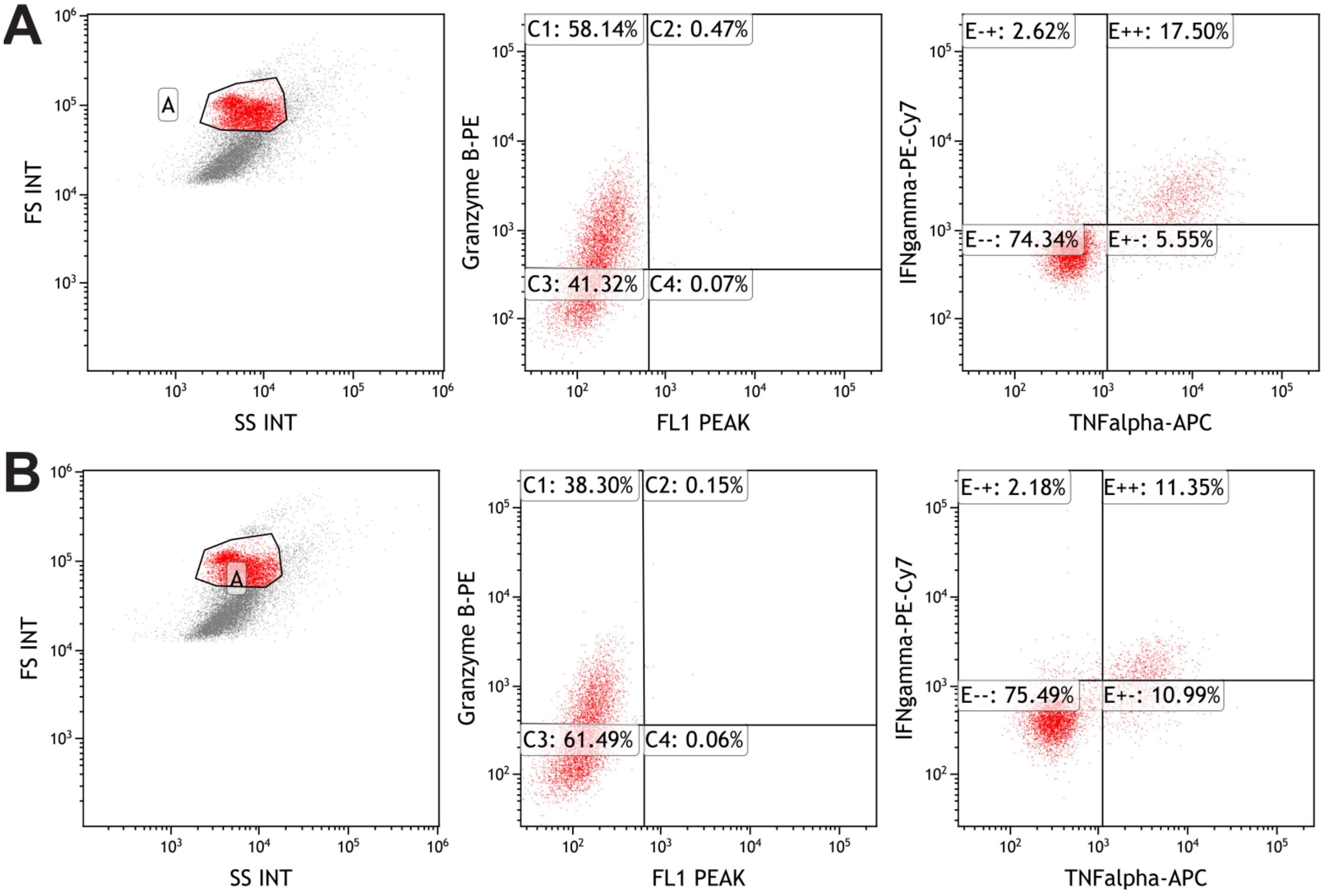

Supplement: Supplementary file 1 [file DataSheet_1.pdf]
